# Drinking water and sanitation: progress in 73 countries in relation to socioeconomic indicators

**DOI:** 10.2471/BLT.15.162974

**Published:** 2015-11-27

**Authors:** Jeanne Luh, Jamie Bartram

**Affiliations:** a The Water Institute, Department of Environmental Sciences and Engineering, University of North Carolina, 4114 McGavran-Greenberg (Campus Box 7431), 135 Dauer Drive, Chapel Hill, North Carolina 27599, United States of America.

## Abstract

**Objective:**

To assess progress in the provision of drinking water and sanitation in relation to national socioeconomic indicators.

**Methods:**

We used household survey data for 73 countries – collected between 2000 and 2012 – to calculate linear rates of change in population access to improved drinking water (*n* = 67) and/or sanitation (*n* = 61). To enable comparison of progress between countries with different initial levels of access, the calculated rates of change were normalized to fall between –1 and 1. In regression analyses, we investigated associations between the normalized rates of change in population access and national socioeconomic indicators: gross national income per capita, government effectiveness, official development assistance, freshwater resources, education, poverty, Gini coefficient, child mortality and the human development index.

**Findings:**

The normalized rates of change indicated that most of the investigated countries were making progress towards achieving universal access to improved drinking water and sanitation. However, only about a third showed a level of progress that was at least half the maximum achievable level. The normalized rates of change did not appear to be correlated with any of the national indicators that we investigated.

**Conclusion:**

In many countries, the progress being made towards universal access to improved drinking water and sanitation is falling well short of the maximum achievable level. Progress does not appear to be correlated with a country’s social and economic characteristics. The between-country variations observed in such progress may be linked to variations in government policies and in the institutional commitment and capacity needed to execute such policies effectively.

## Résumé

**Objectif:**

Évaluer les progrès réalisés dans la fourniture d'eau potable et de services d'assainissement par rapport aux indicateurs socioéconomiques nationaux.

**Méthodes:**

Nous avons utilisé les données d'enquêtes réalisées auprès des ménages dans 73 pays entre 2000 et 2012 pour calculer les taux de changement linéaires de l'accès de la population à un meilleur approvisionnement en eau potable (*n* = 67) et/ou assainissement (*n* = 61). Afin de pouvoir comparer les progrès dans des pays où les niveaux d'accès initiaux différaient, les taux de changement calculés ont été normalisés pour se situer entre -1 et 1. Lors des analyses de régression, nous avons étudié les associations entre les taux de changement normalisés de l'accès de la population et les indicateurs socioéconomiques nationaux: revenu national brut par habitant, efficacité gouvernementale, aide au développement officielle, ressources en eau douce, éducation, pauvreté, coefficient de Gini, mortalité infantile et indice de développement humain.

**Résultats:**

Les taux de changement normalisés indiquaient que la plupart des pays étudiés faisaient des progrès vers l'accès universel à un meilleur approvisionnement en eau potable et à des services d'assainissement. Cependant, seul un tiers des pays montrait un niveau de progression d'au moins la moitié du niveau maximum réalisable. Les taux de changement normalisés ne montraient pas de corrélation avec les indicateurs nationaux que nous avons examinés.

**Conclusion:**

Dans de nombreux pays, les progrès réalisés vers l'accès universel à un meilleur approvisionnement en eau potable et à des services d'assainissement sont bien inférieurs au niveau maximum réalisable. Ces progrès ne montrent pas de corrélation avec les caractéristiques sociales et économiques des différents pays. Les variations observées entre les pays quant à ces progrès peuvent être dues aux variations des politiques gouvernementales ainsi qu’à celles de l'engagement et des capacités institutionnels nécessaires pour appliquer efficacement ces politiques.

## Resumen

**Objetivo:**

Evaluar el progreso del suministro de agua potable y saneamiento en relación con indicadores socioeconómicos nacionales.

**Métodos:**

Se utilizaron los datos de una encuesta domiciliaria realizada en 73 países (recogidos entre los años 2000 y 2012) para calcular la tasa de variación lineal del acceso mejorado de la población al agua potable (n = 67) y/o saneamiento (n = 61). Para poder comparar el progreso entre países con distintos niveles iniciales de acceso, las tasas de variación calculadas se normalizaron para abarcar entre -1 y 1. Se realizaron análisis de regresión en los que se investigó la relación entre las tasas de variación normalizadas del acceso de la población y los indicadores socioeconómicos nacionales: el producto interior bruto per cápita, la eficiencia del gobierno, la ayuda oficial al desarrollo, los recursos de agua dulce, la educación, la pobreza, el coeficiente de Gini, la mortalidad infantil y el índice de desarrollo humano.

**Resultados:**

Las tasas de variación normalizadas indicaron que la mayoría de los países investigados estaban progresando hacia un acceso universal mejorado al agua potable y saneamiento. No obstante, únicamente un tercio de ellos mostró un nivel de progreso equivalente a, al menos, la mitad del máximo del nivel alcanzable. Las tasas de variación normalizadas no mostraban signos de estar relacionadas con ninguno de los indicadores nacionales que se investigaron.

**Conclusión:**

En numerosos países, el progreso conseguido hacia un acceso universal mejorado al agua potable y saneamiento no logra alcanzar el nivel máximo alcanzable. El progreso no parece estar relacionado con las características sociales y económicas de un país. Las variaciones entre países observadas en dicho progreso pueden estar relacionadas con las modificaciones de las políticas gubernamentales y el compromiso y la capacidad necesarios de las instituciones para ejecutar tales políticas de forma eficaz.

## ملخص

**الغرض:**

تقييم مستوى التقدم في توفير مياه الشرب والصرف الصحي فيما يتعلق بالمؤشرات الاجتماعية والاقتصادية الوطنية.

**الطريقة:**

لقد استخدمنا بيانات المسح الأسري لـ 73 دولة – والتي تم جمعها في الفترة ما بين عامي 2000 و2012 – لاحتساب المعدلات الخطية للتغيير في حصول السكان على خدمات مياه الشرب ( *العدد * = 67) و/أو الصرف الصحي المحسّنة ( *العدد * = 61). ولكي يتيسر إجراء مقارنة لمستوى التقدم بين الدول المتفاوتة من حيث المستويات الأولية للخدمات، فقد تمت تسوية معدلات التغيير المحتسبة لتنخفض إلى نطاق يتراوح ما بين –1 و1. وفي تحاليل التحوف، قمنا بالتحقيق في الارتباطات ما بين معدلات التغيير التي تمت تسويتها في حصول السكان على الخدمات والمؤشرات الاجتماعية والاقتصادية الوطنية: الدخل القومي الإجمالي للفرد الواحد، وفعالية أداء الحكومة، والمساعدة الإنمائية الرسمية، وموارد المياه العذبة، والتعليم، والفقر، ومعامل جيني، ووفيات الأطفال، ومؤشر التنمية البشرية.

**النتائج:**

أشارت معدلات التغيير التي تمت تسويتها إلى أن معظم الدول التي تم التحقيق فيها كانت تحقق تقدمًا تجاه تنفيذ الوصول الشامل لخدمات مياه الشرب والصرف الصحي المحسّنة. ومع ذلك، أظهرت ثلث الدول فقط مستوى من التقدم الذي يمثل على الأقل نصف الحد الأقصى للمستوى القابل للتنفيذ. لم تَبدُ معدلات التغيير التي تمت تسويتها مرتبطة مع أي من المؤشرات الوطنية التي قمنا بالتحقيق فيها.

**الاستنتاج:**

في العديد من الدول، إن مستوى التقدم الذي يتم تحقيقه تجاه الوصول الشامل لخدمات مياه الشرب والصرف الصحي المحسّنة ينخفض بشكل كبير عن الحد الأقصى للمستوى القابل للتنفيذ. لا يبدو مستوى التقدم مرتبطًا مع الخصائص الاجتماعية والاقتصادية للدولة. وقد تكون الاختلافات الملحوظة بين كل بلد في مثل هذا المستوى من التقدم مرتبطة بالاختلافات في السياسات الحكومية والتزام المؤسسات والقدرات اللازمة لتنفيذ مثل هذه السياسات على نحو فعال.

## 摘要

**目的:**

旨在评估饮用水和卫生设施供应的进展与国家社会经济指标的相关性。

**方法:**

我们使用了 73 个国家在 2000 年至 2012 年之间收集的家庭调查数据，以计算人们获得改善的饮用水 (*n *= 67) 和/或卫生设施 (*n* = 61) 的线性变化率。 为了对不同初始水平国家之间的进展进行比较，将变化的计算率规范化为 -1 至 1。在回归分析中，我们研究了人们获得的规范化的变化率和国家社会经济指标的相关性： 人均国民收入、政府效率、官方发展援助、淡水资源、教育、扶贫、基尼系数、儿童死亡率和人类发展指数。

**结果:**

规范化的变化率说明大多数参与调查的国家不断取得进步，正逐步实现普及改善饮用水和卫生设施。 然而，只有大约三分之一的国家达到了最高进步水平的一半以上。 规范化的变化率似乎并未与我们所调查的任何一项国家指标相关。

**结论:**

在许多国家，普及改善饮用水和卫生设施的进展远低于可达到的最高水平。 进展似乎并不与一个国家的社会和经济特点相关。 进展呈现的国家之间的差异可能与政府政策和机构承诺的差异以及有效地执行这种政策所需的能力相关。

## Резюме

**Цель:**

Оценить прогресс в снабжении питьевой водой и в вопросах санитарии в связи с национальными социально-экономическими показателями.

**Методы:**

Были использованы данные опроса семей в 73 странах, полученные в период между 2000 и 2012 годами, и по этим данным были рассчитаны линейные показатели изменения доступа населения к питьевой воде улучшенного качества (n = 67) и (или) к услугами санитарии (n = 61). Чтобы можно было сравнивать прогресс для стран с различным начальным уровнем такого доступа, все расчетные значения показателей изменения были нормализованы таким образом, чтобы новые значения находились в диапазоне от -1 до 1. В ходе регрессионного анализа была изучена связь между нормализованными показателями изменения доступа населения и национальными социально-экономическими показателями: валовым национальным доходом на душу населения, эффективностью деятельности правительства, официальной помощью в целях развития, запасами свежей воды, уровнями образования и бедности, коэффициентом Джини, детской смертностью и индексом развития человеческого потенциала.

**Результаты:**

Судя по нормализованным показателям изменения, большинство изученных стран делают успехи на пути к всеобщей доступности санитарных услуг и воды улучшенного качества. Однако примерно в трети случаев уровень прогресса был по меньшей мере вполовину ниже максимально достижимого значения. Как оказалось, нормализованные показатели изменений не коррелируют ни с одним из проверенных нами национальных показателей.

**Вывод:**

Во многих странах прогресс, наблюдаемый в обеспечении всего населения санитарными услугами и питьевой водой улучшенного качества, намного ниже максимально достижимого уровня. Не было выявлено корреляции между таким прогрессом и социально-экономическими характеристиками соответствующих стран. Наблюдаемые на уровне отдельных стран различия в таком прогрессе могут быть связаны с различными государственными подходами, а также с уровнем внимания, уделяемого этим задачам со стороны учреждений, и с наличием потенциала для эффективного осуществления соответствующих стратегических планов.

## Introduction

The United Nations recognizes the basic human right to water and sanitation.^1^^,^^2^ Accordingly, the international community, through the recent adoption of the Sustainable Development Goals (SDGs), has made a commitment to achieve universal and equitable access to safe drinking water and adequate sanitation by 2030.^3^ The SDGs build on the Millennium Development Goal (MDG) target^4^ to halve, between 1990 and 2015, the proportion of the population without access to safe water and basic sanitation. During the MDG period, some countries have made substantial progress, while others have stagnated.^5^ The national characteristics that may enhance or hinder progress on water and sanitation are poorly understood. For example, external finance should make it easier for governments to improve drinking water and sanitation coverage. While a positive correlation between aid received and improvements in such coverage has been observed in some studies,^6^^,^^7^ other studies have not detected such a relationship.^8^^–^^11^ The differing results may be due to limitations in the methods used^8^ and/or the choice of indicator used to measure progress. Progress has been measured as population access to improved drinking water and sanitation – or the change in such access over a specified period. However, changes in population access are not necessarily comparable across different countries because, as a country approaches universal access, it becomes increasingly difficult to reach those who still lack access.

The aim of the present study is to determine whether progress in improving access to improved drinking water and sanitation, achieved by countries between 2000 and 2012, is associated with national socioeconomic characteristics. We used a new indicator of progress – the normalized rate of change in access – to allow countries to be compared, regardless of their initial coverage levels.

## Methods

### Data sources

We obtained estimates of the percentage of national populations with access to improved sanitation and water – for various years between 2000 and 2012 – from the 2013 Country Files of the Joint Monitoring Programme for Water Supply and Sanitation^12^ – which were the most up-to-date information available at the time of analyses. This World Health Organization/United Nations Children’s Fund programme compiles the results of nationally representative surveys, including Demographic and Health Surveys, Multiple Indicator Cluster Surveys, World Health Surveys and national censuses. We considered only data from 2000 onwards to reflect the progress countries made since the MDGs were set in the year 2000.

We included shared toilet facilities in our improved-sanitation category because data for both shared sanitation and total improved sanitation including shared sanitation – i.e. the two data sets needed to investigate total improved sanitation excluding shared sanitation – were only available for four of our study countries. The Joint Monitoring Programme currently discounts shared sanitation from total improved sanitation by applying a fixed ratio for each country.^13^ However, since these ratios are based on data that may have been collected before 2000 and, for some countries, are based on a single data point, we decided not to use them – or any other similar correction factor – in our analyses. We included countries with at least five data points that covered at least three different years. Multiple survey data points from any one year were treated independently.

### Indicator of progress

To compare countries with differing initial levels of population access to improved sanitation and water, we defined the progress of country *i* as its normalized rate of change in access:

$$normalized\;rate_{i,j}=\frac{rate_{i,j}\;–\;min.\;rate_{j}}{max.\;rate_{j}\;–\;min.\;rate_{j}}$$ (1)

where *normalized rate_i,j_* is the normalized rate of change for country *i* that had a baseline coverage level *j* in the year 2000; *rate_i,j_* is the absolute rate of change for country *i* at coverage level *j*; *max. rate_j_* is the maximum rate achievable by any country at coverage level *j* (based on historical data, see below) and the *min. rate_j_* is set at zero (no progress). Each country’s absolute rate of change was calculated from the earliest available year (2000 in most cases) using linear regression.

We determined values for the maximum rate achievable at each coverage level using the frontier approach.^14^^,^^15^ Historical absolute rates of change for all countries were plotted as a function of the national coverage level for the year 2000. For countries that had survey data for 2000, we used those values for national coverage level. For countries that did not have surveys for 2000, we used estimates from the Joint Monitoring Programme.^12^ The best-performing countries (which we refer to as frontier points) delineate an upper boundary or frontier against which the performance of the other countries can be compared. We used the frontier efficiency analysis package^16^ in R software^17^ to identify frontier points.

A polynomial curve was fitted through the frontier points to obtain the frontier curve – with the requirement that the curve must pass through the point corresponding to 100% coverage and 0% increase in coverage per year. The frontier curve allowed the maximum achievable rates of improvement in water and sanitation coverage to be estimated for all countries, depending on their initial level of coverage (Table 1). Using the estimated maximum achievable rates and Equation 1, we obtained the normalized rates of change for our study countries. The requirement that the frontier curve must pass through the point corresponding to 100% coverage and 0% increase in coverage per year meant that the frontier curve – which is the fitted polynomial equation – sometimes fell below a frontier point. This resulted in a normalized rate greater than 1 for some frontier countries. We assigned a normalized rate of 1 to all such countries. Similarly, for countries in which we found access to improved drinking water and sanitation to be decreasing, we limited the negative normalized rate to –1. All of the normalized rates we report therefore fall between –1 and 1.

**Table 1:** Population access to improved water and sanitation, linear rates of change and corresponding normalized rates of change, 73 countries, 2000–2012

Country	Drinking water							Sanitation					
	No. of data points	Years	*R*^2a^	2000 coverage (%)	Rate of change			No. of data points	Years	*R*^2a^	2000 coverage (%)	Rate of change	
					Absolute (%/year)	Normalized						Absolute (%/year)	Normalized
Albania	5	2000–2009	0.30	97.1	–0.19	–0.57		5	2000–2009	0.34	90.5	0.47	0.43
Armenia	13	2000–2011	0.49	95.3	0.50	0.93		0	–	–	–	–	–
Bangladesh	10	2000–2011	0.76	76.2	0.82	0.42		10	2000–2011	0.83	68.4	1.51	0.77
Belize	5	2000–2009	0.65	89.3	0.85	0.77		5	2000–2009	0.78	90.9	0.50	0.47
Benin	6	2001–2009	0.17	66.1^b^	0.57	0.25		6	2001–2009	0.90	9.0^b^	1.45	0.83
Bolivia (Plurinational State of)	10	2000–2009	0.74	80.1	1.00	0.57		11	2000–2009	0.47	58.5	1.13	0.50
Botswana	9	2000–2008	0.34	95.4	0.20	0.37		8	2000–2008	0.48	60.3	0.88	0.40
Brazil	21	2000–2011	0.54	91.8	0.30	0.34		20	2000–2011	0.78	71.6	0.96	0.51
Burkina Faso	11	2003–2010	0.35	59.9^b^	1.08	0.45		0	–	–	–	–	–
Cabo Verde								6	2000–2010	0.86	38.5	1.38	0.44
Cambodia	8	2000–2011	0.51	46.8	1.77	0.72		8	2000–2011	0.92	19.2	1.95	0.64
Cameroon	5	2000–2007	0.35	59.4	1.03	0.42		5	2000–2007	0.06	62.1	–0.38	–0.17
Chad	5	2000–2010	0.47	46.3	0.15	0.06		5	2000–2010	0.02	13.1	0.06	0.02
Chile	5	2000–2009	0.89	94.9	0.47	0.81		5	2000–2009	0.92	92.0	0.57	0.59
Colombia	0	–	–	–	–	–		5	2000–2010	0.68	84.2	1.12	0.76
Costa Rica	11	2000–2011	0.16	95.2	0.16	0.29		11	2000–2011	0.67	94.1	0.34	0.44
Côte d’Ivoire	5	2000–2008	0.31	80.6	–0.92	–0.53		5	2000–2008	0.02	40.6	0.20	0.06
Democratic Republic of the Congo	0	–	–	–	–	–		5	2001–2010	0.44	22.6^b^	–1.41	–0.44
Dominican Republic	0	–	–	–	–	–		6	2000–2010	0.01	92.2	0.03	0.03
Egypt	5	2000–2008	0.83	97.3	0.07	0.23		5	2000–2008	0.97	97.3	0.76	1.0
Estonia	5	2000–2003	0.25	98.9	–0.06	–0.49		10	2000–2004	0.00	98.9	0.01	0.05
Ethiopia	6	2000–2011	0.98	26.2	2.28	1.0		7	2000–2011	0.89	11.9	2.20	0.97
Georgia	0	–	–	–	–	–		6	2000–2010	0.70	97.5	–0.22	–0.59
Ghana	9	2000–2008	0.87	66.5	2.29	1.0		9	2000–2008	0.65	59.8	1.96	0.88
Guatemala	7	2000–2009	0.19	86.4	0.41	0.31		6	2000–2009	0.58	80.2	0.66	0.40
Guinea	5	2002–2007	0.20	63.2^b^	1.13	0.48		5	2002–2007	0.75	14.1^b^	1.96	0.76
Guyana	5	2000–2009	0.50	88.8	0.50	0.44		5	2000–2009	0.52	86.3	0.48	0.35
Honduras	14	2001–2011	0.75	80.8^b^	0.75	0.43		7	2001–2011	0.93	64.5^b^	2.06	0.99
India	9	2000–2011	0.34	83.2	0.44	0.28		9	2000–2011	0.97	31.5	1.48	0.45
Indonesia	12	2001–2010	0.56	77.7^b^	1.04	0.55		0	–	–	–	–	–
Iraq	5	2000–2011	0.33	82.6	0.87	0.54		0	–	–	–	–	–
Jamaica	10	2000–2009	0.00	92.4	–0.00	–0.00		10	2000–2009	0.04	97.0	0.03	0.07
Jordan	5	2002–2010	0.10	96.7^b^	–0.14	–0.38			–	–	–	–	–
Kenya	7	2000–2010	0.27	52.9	0.70	0.28		7	2000–2010	0.17	50.3	0.51	0.20
Lao People's Democratic Republic	6	2000–2012	0.85	45.4	2.21	0.90		9	2000–2012	0.89	26.6	3.16	0.95
Lesotho	6	2000–2009	0.00	76.2	–0.04	–0.02		5	2000–2009	0.28	32.8	0.25	0.08
Liberia	6	2000–2011	0.30	60.5	1.11	0.46		6	2000–2011	0.15	30.3	0.71	0.22
Madagascar	9	2000–2011	0.78	33.6	1.33	0.56		8	2000–2011	0.15	32.0	0.23	0.07
Malawi	13	2000–2011	0.55	65.5	1.43	0.61		11	2000–2011	0.47	74.6	0.90	0.50
Mali	5	2001–2010	0.93	45.5^b^	3.47	1.0		6	2001–2010	0.20	18.2^b^	0.32	0.11
Mauritania	5	2000–2007	0.04	36.8	0.42	0.17		5	2000–2007	0.84	27.1	1.51	0.42
Mexico	9	2000–2010	0.52	87.2	0.78	0.61		11	2000–2010	0.55	81.7	1.06	0.67
Mongolia	5	2000–2007	0.77	66.8	1.54	0.67		0	–	–	–	–	–
Morocco	10	2000–2007	0.88	74.0	1.29	0.63		0	–	–	–	–	–
Mozambique	5	2003–2009	0.58	41.1^b^	1.48	0.61		8	2001–2009	0.45	14.1^b^	1.10	0.43
Myanmar	0	–	–	–	–	–		6	2000–2010	0.43	71.4	1.28	0.68
Namibia	5	2000–2007	0.21	77.6	0.80	0.43			–	–	–	–	–
Nepal	7	2001–2011	0.11	77.4^b^	0.32	0.17		9	2000–2011	0.73	28.5	2.19	0.66
Nicaragua	5	2001–2006	0.48	80.0^b^	0.56	0.32		0	–	–	–	–	–
Niger	5	2000–2008	0.08	43.3	0.44	0.18		0	–	–	–	–	–
Nigeria	10	2000–2011	0.55	54.0	0.77	0.31		10	2000–2011	0.44	63.9	–1.02	–0.49
Pakistan	12	2002–2009	0.02	88.3^b^	0.15	0.13		9	2002–2008	0.86	37.4^b^	2.30	0.73
Paraguay	6	2000–2004	0.23	77.0	0.95	0.49		5	2000–2004	0.12	65.2	0.87	0.42
Peru	6	2000–2009	0.04	83.0	0.14	0.09		6	2000–2009	0.01	68.8	0.09	0.05
Philippines	6	2000–2008	0.21	91.3	–0.15	–0.16		6	2000–2008	0.51	81.4	0.52	0.33
Republic of Korea	7	2000–2006	0.99	93.5	0.50	0.69		0	–	–	–	–	–
Republic of Moldova	13	2000–2010	0.40	93.0	0.35	0.46		13	2000–2010	0.73	84.4	0.88	0.60
Rwanda	11	2000–2010	0.00	68.7	0.06	0.03		10	2000–2010	0.84	52.0	2.56	1.0
Samoa	5	2001–2011	0.66	93.3^b^	0.89	1.0		0	–	–	–	–	–
Senegal	7	2000–2011	0.66	67.3	0.77	0.34		8	2000–2011	0.46	56.3	0.82	0.35
Sierra Leone	5	2003–2010	0.89	46.8^b^	1.28	0.52		7	2000–2011	0.33	37.0	0.67	0.21
South Africa	7	2000–2008	0.83	85.1	0.71	0.49		8	2000–2008	0.13	74.3	0.58	0.32
Sri Lanka	7	2000–2010	0.70	74.6	1.25	0.62		5	2000–2010	0.61	89.2	1.06	0.90
Swaziland	5	2000–2010	0.59	51.8	1.21	0.49		6	2000–2010	0.48	68.4	0.71	0.36
Tajikistan	6	2000–2009	0.18	59.1	0.54	0.22		5	2000–2007	0.62	59.1	0.80	0.35
Thailand	5	2000–2006	0.20	93.2	0.26	0.35		5	2000–2006	0.16	99.1	–0.04	–0.27
Timor-Leste	6	2001–2010	0.52	54.3^b^	1.35	0.55		6	2001–2010	0.02	37.4^b^	0.17	0.05
Uganda	10	2001–2010	0.77	56.8^b^	1.61	0.66		11	2000–2010	0.51	48.1	0.68	0.25
United Republic of Tanzania	10	2000–2011	0.00	55.5	–0.03	–0.01		10	2000–2011	0.70	14.0	0.88	0.34
Uruguay	5	2003–2011	0.00	97.9^b^	–0.01	–0.03		5	2003–2011	0.73	96.7^b^	–0.16	–0.34
Viet Nam	8	2000–2011	0.82	78.8	1.20	0.66		8	2000–2011	0.52	61.2	1.20	0.55
Zambia	6	2002–2010	0.61	53.6^b^	1.04	0.42		7	2000–2010	0.02	56.9	0.11	0.05
Zimbabwe	5	2003–2011	0.09	79.6^b^	–0.29	–0.17		5	2003–2011	0.16	40.4^b^	–0.34	–0.11

^a^ A measure of goodness of fit for the linear regression.

^b^ Estimates from the Joint Monitoring Programme for Water Supply and Sanitation.^12^

### Regression analyses

We used regression analyses to investigate the relationship between progress in water and sanitation and the following national socioeconomic indicators: (i) gross national income per capita – in current United States dollar (US$) values that had been derived using the Atlas method;^18^ (ii) government effectiveness;^19^ (iii) the per-capita level of official development assistance for sanitation and water – calculated, in constant 2011 values, by dividing the total assistance disbursed from all donors^20^ by the total population;^21^ (iv) the volume of renewable internal freshwater resources per-capita;^18^ (v) the percentage of the female population older than 25 years that had completed secondary education;^22^ (vi) the percentage of the population with a daily income of less than US$ 1.25;^18^ (vii) the Gini coefficient;^18^ (viii) the mortality rate among children younger than five years;^18^ and (ix) the human development index – a composite index reflecting life expectancy, education and income.^23^ For each indicator and country, we used the value for the year 2000 or, if that value was not available, that for the closest available year.

We initially considered data from the World Health Organization’s Global Analysis and Assessment of Sanitation and Drinking Water reports, which provide policy and economic indicators such as the per-capita budget for drinking water and sanitation from the year 2010^24^ and per-capita expenditure on sanitation and water in the year 2014.^25^ However, as these data relate to time periods that are at least 10 years off from our target year of 2000 – and indicators such as expenditures per capita may vary substantially from year to year – we decided not to include them in our analyses.

Several of the nine national characteristics we investigated were highly correlated. We therefore used principal components analysis on the nine national indicators to obtain uncorrelated synthetic independent variables (Table 2). However, based on the Kaiser criterion, we only used the three synthetic variables that gave eigenvalues greater than 1 – which together accounted for 76% of the variance in the data observed – in our regression analyses. Backward stepwise regression – with *P*-values of 0.05 and 0.10 for the addition and deletion of variables, respectively – was also used to identify a subset of the three synthetic independent variables for the regression analyses.

**Table 2:** Results of principal component analysis based on nine national socioeconomic indicators for all 73 study countries

Indicator	Component								
	1	2	3	4	5	6	7	8	9
Gini coefficient	0.157	0.660	0.353	0.217	0.295	–0.445	–0.165	–0.230	0.050
Proportion of population with daily income below US$ 1.25^a^	–0.407	0.174	–0.011	–0.032	0.443	0.322	–0.513	0.490	0.005
Mortality rate among children aged < 5 years	–0.434	0.175	0.127	–0.015	0.104	–0.066	0.693	0.249	0.455
Per-capita volume of renewable internal freshwater resources	0.088	0.576	–0.523	0.357	–0.299	0.395	0.115	0.011	–0.036
Per-capita gross national income	0.440	0.124	0.157	–0.116	–0.167	–0.186	0.154	0.755	–0.313
Government effectiveness	0.316	0.051	0.555	–0.059	0.155	0.709	0.190	–0.153	–0.013
Per-capita level of official development assistance for sanitation and water	–0.169	–0.268	0.365	0.806	–0.282	0.000	–0.110	0.156	0.013
Percentage of the female population older than 25 years that had completed secondary education	0.280	–0.264	–0.328	0.396	0.697	–0.038	0.282	0.037	–0.142
Human development index	0.462	–0.107	–0.108	0.021	–0.027	0.013	–0.254	0.162	0.820
Eigenvalue	4.395	1.318	1.089	0.896	0.597	0.391	0.194	0.091	0.029
Proportion	0.488	0.146	0.121	0.010	0.066	0.044	0.022	0.010	0.003
Cumulative	0.488	0.635	0.756	0.855	0.922	0.965	0.987	0.997	1.000

US$: United States dollars.

^a^ As defined by the World Bank.^18^

Univariate and multivariate regression analyses were performed in Stata version 12 (Stata Corp. LP, College Station, United States of America). We ran models using the data from all of our study countries and, separately, using only the data from those study countries that had no armed conflict between 2000 and 2012.^26^ While regression results do not necessarily provide information on causality, a predictive empirical model could be useful in estimating the progress towards universal access in countries where sanitation and water data are not available. We analysed the relationship between the normalized rates of change and the nine national indicators that we investigated, as independent variables, using a linear model:

$$\begin{array}{l} normalized\;rate=\\ \beta_{1}x_{1}\;+\;\beta_{2}x_{2}\;+\;\ldots \;+\;\beta_{i}x_{i}\;+\;constant \end{array}$$ (2)

and a fractional logistic model:

$$\begin{array}{l} \log\frac{normalized\;rate}{\text{1}\;–\;normalized\;rate}=\\ \beta_{1}x_{1}\;+\;\beta_{2}x_{2}\;+\;\ldots \;+\;\beta_{i}x_{i}\;+\;constant \end{array}$$ (3)

where *β_1_* to *β_i_* are the fitted model coefficient values and *x_1_* to *x_i_* are the independent variables. Countries with negative normalized rates were excluded from the fractional logistic regressions because, for these, the output parameter must lie between 0 and 1. These regressions therefore focused only on countries that had made progress in increasing access to improved sanitation and water. We re-ran the models using the synthetic independent variables.

### Country pairings

We selected countries where, despite similar initial coverage, we observed marked differences in progress. To understand possible reasons for these differences in progress, we chose discordant pairs of countries within the same geographic region and with similar characteristics – as defined by the country clusters of Onda et al.^27^ – and compared their national socioeconomic indicators.

## Results

National access to improved sanitation and water in the year 2000 and historical absolute rates of change are shown in Table 1. Relatively few relevant data were available from high-income countries that are approaching or have already achieved universal access. High-income countries were therefore not well represented in our analyses. The absolute rates of change in access to improved drinking water and sanitation ranged from –0.9% to 3.5% per year (67 countries) and from –1.4% to 3.2% per year (61 countries), respectively.

**Fig. 1:**
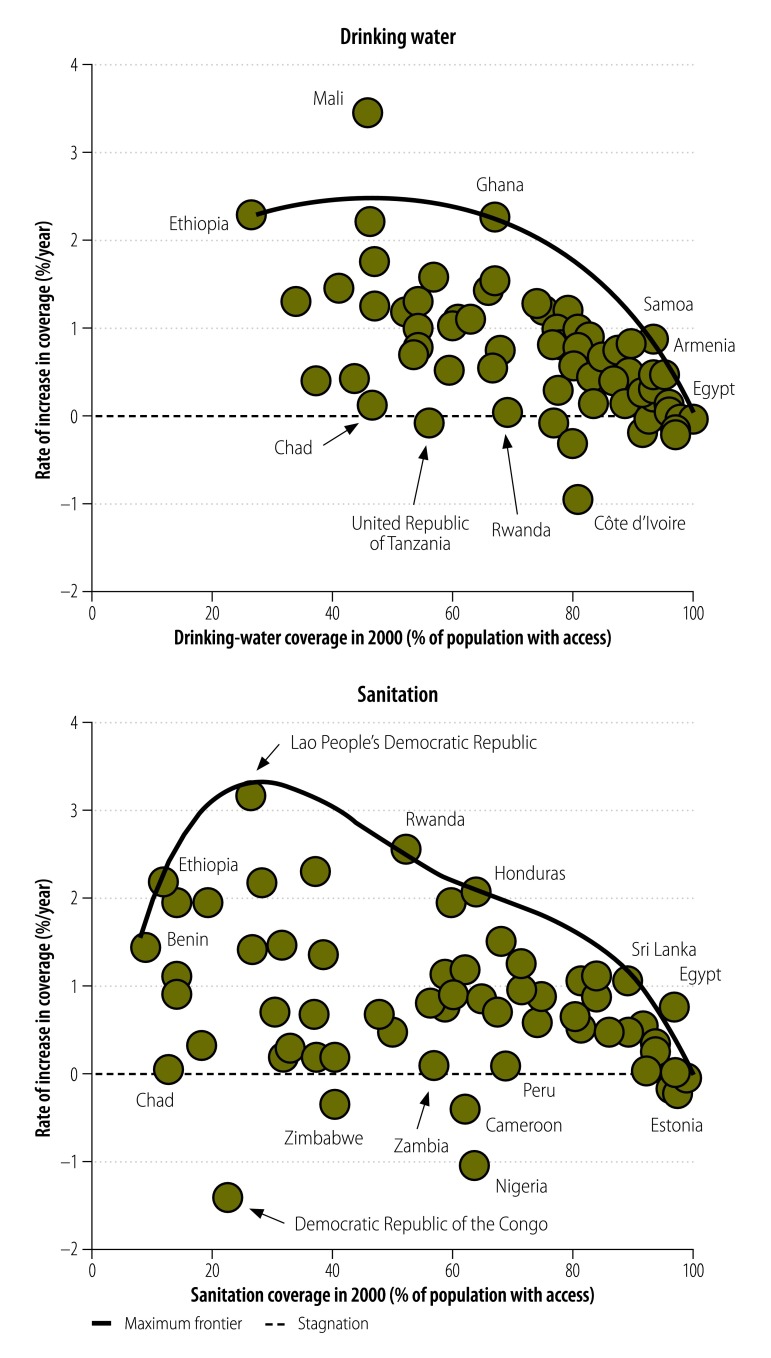
Historical absolute rates of change in access to sanitation and drinking water, 2000–2012

Notes: Rates were calculated for 2000–2012, and are shown as a function of the national coverage in the year 2000. Each data point represents a different country – 67 for water and 61 for sanitation – but only the names of some of the countries with particularly good or poor rates of change are shown.

The frontier curves used to calculate the maximum rates of change in Equation 1 – shown as solid lines in Fig. 1 – were constructed using five frontier points for water – based on data from Armenia, Egypt, Ethiopia, Ghana and Samoa – and eight frontier points for sanitation – based on data from Benin, Egypt, Estonia, Ethiopia, Honduras, Lao People's Democratic Republic, Rwanda and Sri Lanka. For water, Mali was identified as an outlier^28^^,^^29^ and not used to construct the frontier curve. The frontier curves for both sanitation and water indicate decreases in the maximum achievable rate of change as countries approach 100% coverage.

While positive and negative absolute rates indicate countries with increasing and decreasing coverage, respectively, only the normalized rates in Table 1 should be used to compare the performances of the study countries. These normalized rates indicate that, over our study period and for both water and sanitation, only about one in every three of our study countries progressed at a rate that was at least half of their maximum achievable rate – i.e. they had normalized rates that were greater than 0.5. Among the countries with relevant data, 20 (30%) of 67 had normalized rates for water that fell below 0.25 and 21 (34%) of 61 had the same low normalized rates for sanitation.

**Fig. 2:**
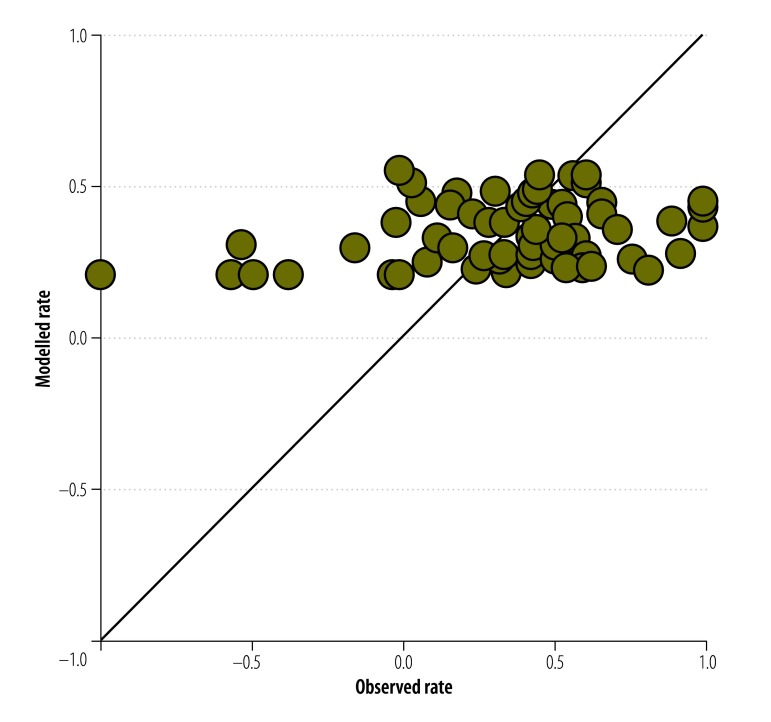
Observed and modelled normalized rates of change in access to drinking water in 63 countries, 2000–2012

Notes: The plot shows estimates from a linear regression in which the proportion of the population with a daily income below 1.25 United States dollars was used as the independent variable. The solid line indicates a perfect match between the observed rates and the modelled estimates.

**Fig. 3:**
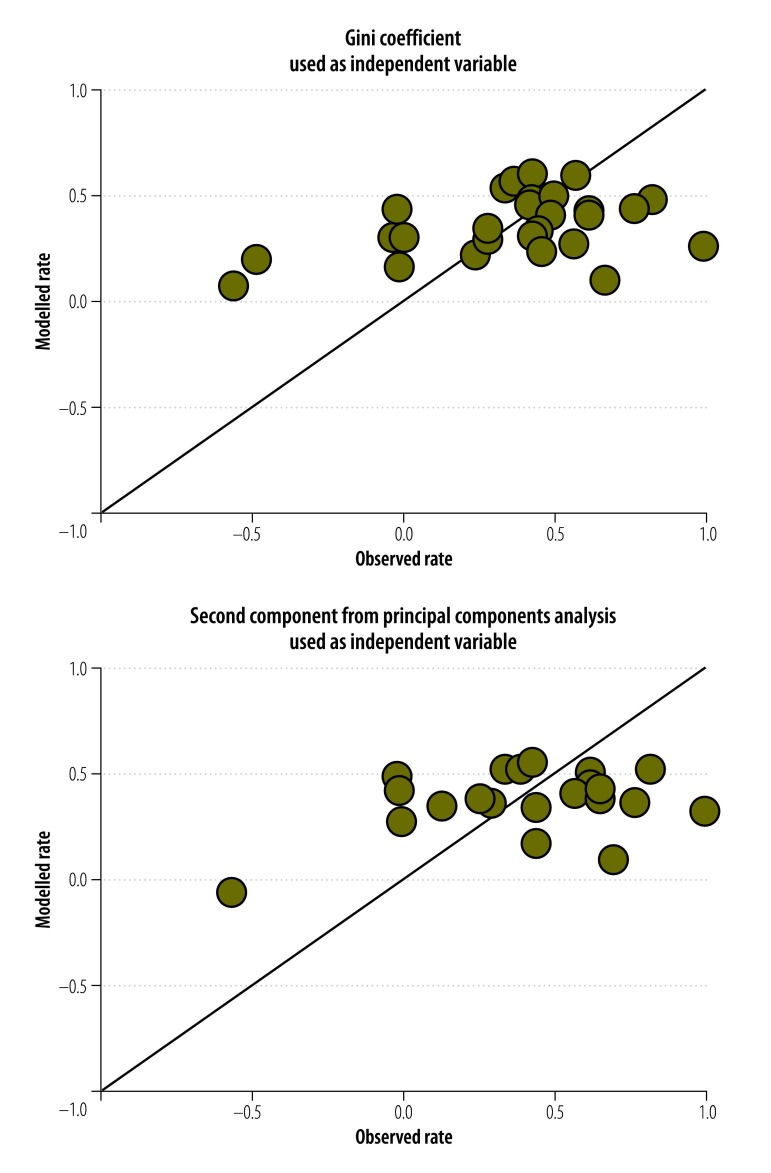
Observed and modelled normalized rates of change in access to drinking water in 27 countries with no armed conflict, 2000–2012

Notes: The plots show estimates from linear regressions, with either the Gini coefficient or the second component from principal components analysis used as the independent variable. The solid lines indicate a perfect match between the observed rates and the modelled estimates.

Using the normalized rate as our indicator of progress, only two univariate regression models for access to drinking water – and no models for sanitation – were statistically significant overall (*P* ≤ 0.05; Table 3). However, the model fit was poor (adjusted *R^2^* < 0.2) and Fig. 2 and Fig. 3 show the poor agreement between the observed and modelled estimates.

**Table 3:** Regression model results for the associations between normalized rates of change in improved water and sanitation coverage and socioeconomic indicators

Model type, coverage type^a^	Independent variable	Regression type	Inclusion of countries with armed conflict?	*n*	Coefficient	SE (95% CI)
**Univariate**						
Water	Poverty^b^	Linear	Yes	63	0.004	0.0018 (0.0004 to 0.0077)
Water	Gini coefficient	Linear	No	27	0.015	0.0068 (0.0010 to 0.0291)
**Multivariate**						
Sanitation	Component 2^c^	Linear	Yes	50	–0.0903	0.0449 (–0.1801 to –0.00004)
Water	Component 2^c^	Linear	No	23	0.124	0.0573 (0.0048 to 0.2433)

CI: confidence interval; SE: standard error.

^a^ Only the results for regressions that gave *P*-values of no greater than 0.05 are shown.

^b^ Proportion of the population with daily income below 1.25 United States dollars.

^c^ Second component obtained from principal components analysis (Table 2).

**Fig. 4:**
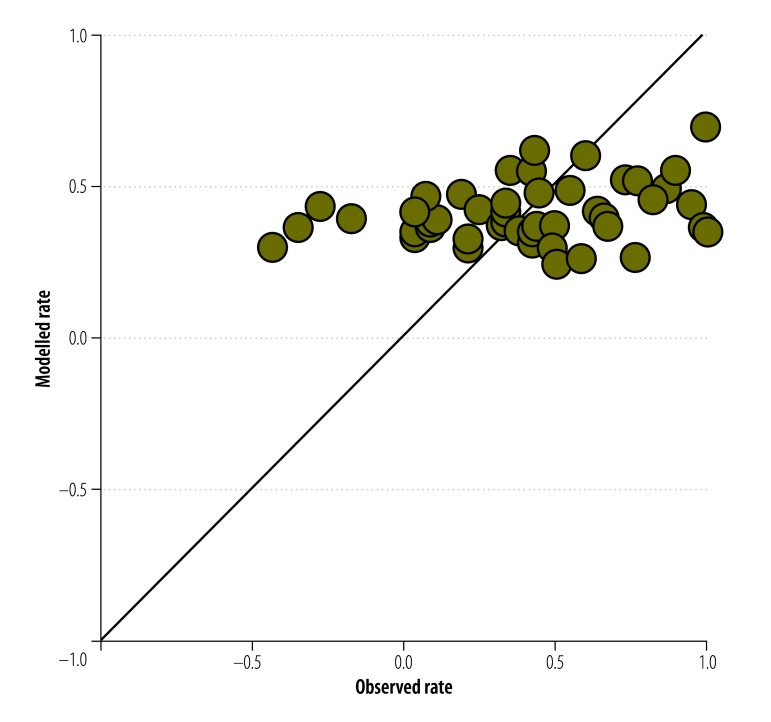
Observed and modelled normalized rates of change in access to sanitation in 50 countries, 2000–2012

Notes: The plot shows estimates from a linear regression in which the second component from a principal components analysis was used as the independent variable. The solid line indicates a perfect match between the observed rates and the modelled estimates.

Multivariate regression with the three synthetic independent variables resulted in two models – i.e. one for water and one for sanitation – that were statistically significant (Table 3). Again, however, there was poor agreement between the observed and the modelled estimates (Fig. 3 and Fig. 4).

Overall, our results show no correlation between the normalized rates of change in the improvement of access to drinking water or sanitation and any of the nine national indicators that we investigated or any of the principal components obtained from these indicators. A similar lack of correlation was observed when the analyses were performed using the most recent data available for each of the nine national indicators (available from the corresponding author).

An analysis of the illustrative pairs of countries with differing progress indicate that no single indicator was consistently associated with progress in coverage for water or sanitation (Table 4 or Table 5, respectively, available at: http://www.who.int/bulletin/volumes/94/2/15-162974).

**Table 4:** Comparison of selected national socioeconomic indicators in pairs of countries with differing progress in drinking water coverage, 2000–2012

Characteristic	Pair 1			Pair 2			Pair 3	
	Egypt	Jordan		Philippines	Thailand		United Republic of Tanzania	Uganda
Country cluster^a^	3	3		4	4		5	5
Geographical area	Eastern Mediterranean	Eastern Mediterranean		South-east Asia	South-east Asia		East Africa	East Africa
Normalized rate	0.23	–0.38		–0.16	0.35		–0.01	0.66
Initial coverage (%)	97.3	96.7		91.3	93.2		55.5	56.8
Per-capita gross national income (current US$)	1471	1797		1048	1959		297	264
Per-capita level of official development assistance for sanitation and water (constant 2011 US$)	1.91	12.4		0.15	0.11		1.04	1.44
Per-capita volume of renewable internal freshwater resources (m^3^)	26.4	135.4		5917	3519		2346	1503
Gini coefficient^b^	32.8	36.4		46.1	42.8		34.6	43.1
Government effectiveness^c^	–0.16	–0.01		–0.14	0.20		–0.42	–0.38

US$: United States dollars.

^a^ As defined by Onda et al.^27^

^b^ The lower the Gini coefficient, the greater the equality.

^c^ As defined by the World Bank.^19^ The higher the value, the stronger the performance of governance.

**Table 5:** Comparison of selected national socioeconomic indicators in pairs of countries with differing progress in sanitation coverage, 2000–2012

Characteristic	Pair 1			Pair 2			Pair 3	
	Costa Rica	Dominican Republic		Paraguay	Peru		Kenya	Rwanda
Country cluster^a^	3	3		4	4		5	5
Geographical area	Central America and the Caribbean	Central America and the Caribbean		South America	South America		East Africa	Central/East Africa
Normalized rate	0.44	0.03		0.42	0.05		0.20	1.0
Initial coverage (%)	94.1	92.2		65.2	68.8		50.3	52.0
Per-capita gross national income (current US$)	3704	2596		1346	2052		421	233
Per-capita level of official development assistance for sanitation and water (constant 2011 US$)	0.13	0.61		0.07	0.65		0.85	0.84
Per-capita volume of renewable internal freshwater resources (m^3^)	27 456	2350		16 872	60 457		627	1057
Gini coefficient^b^	46.5	52.0		57.0	50.8		42.5	51.5
Government effectiveness^c^	0.25	–0.33		–1.17	–0.09		–0.54	–0.65

US$: United States dollars.

^a^ As defined by Onda et al.^27^

^b^ The lower the Gini coefficient, the greater the equality.

^c^ As defined by the World Bank.^19^ The higher the value, the stronger the performance of governance.

## Discussion

The historical absolute rates of change in access to sanitation and water varied greatly at all coverage levels. Over our study period, most countries increased their sanitation and water coverage. Ethiopia and the Lao People's Democratic Republic, for example, showed absolute rates of change – in access to both drinking water and sanitation – in excess of  2.2% per year. Although several countries were found to have decreasing sanitation or water coverage, only one of the countries we investigated – Zimbabwe – showed decreasing coverage for both sanitation and water. We determined normalized rates of change to compare progress between countries. For example, while both Kenya and South Africa had an absolute rate of change of 0.70% per year for water, the corresponding normalized rate for Kenya (0.28) was markedly lower than that for South Africa (0.49) – indicating that South Africa was making greater progress than Kenya.

National socioeconomic characteristics may not be primary determinants of progress in access to water and sanitation. For example, from the illustrative country pairings, Peru might be expected to make better progress than Paraguay – since, per capita, Peru has the greater gross national income, external financial assistance and renewable freshwater resources. However, the normalized rates that we calculated indicate that, over our study period, Paraguay was making good progress whereas Peru was making no progress. Factors other than the nine national indicators we investigated are probably more important than those indicators in determining progress towards universal access. For example, government policies – and variation in the provision of the institutional commitment and capacity needed to execute such policies effectively – may be important determinants of such progress. The lack of association we observed between progress and per-capita level of official development assistance is consistent with previous studies^8^^–^^11^ – although these earlier investigations used different measures of progress and varied in their scale, from global to city level.

Our study has several limitations. We calculated absolute rates of change in coverage of water and sanitation using a linear fit to the data points – even though progress may have been nonlinear during our study period. This may affect the estimated rates of change, the identification of frontier countries and consequently, the frontier curve, the corresponding maximum rates and the normalized rates. Household surveys used as our data sources did not include extra-household settings – e.g. educational institutions, workplaces and health-care settings – and therefore did not represent sanitation and water access for all dimensions of society. Neither did the surveys distinguish between the different levels of improved sanitation or water services – e.g. between a household tap and a community hand pump or between a pit latrine and a sewer connection. Furthermore, inequalities in access often exist. Coverage and service levels tend to be relatively poor among marginalized and vulnerable groups and this may not be captured by national surveys. Identification of the disadvantaged groups in each country is needed so that progress among these groups can be compared with that in the general population.

With respect to our regression analyses, we recognize that the variables we used as national economic indicators may not accurately reflect the levels of investment in sanitation and water. For example, such indicators exclude the many household investments, particularly in sanitation, that occur in developing countries. In addition, the data for the nine national indicators that we investigated were for a single year and did not cover all of our 2000–2012 study period. Alternatives to linear and logistic regression, such as generalized additive models, need to be tested in future studies.

Use of normalized rates allowed countries to be compared regardless of their coverage level, aligns with the human rights principle of progressive realization and could be extended to measure progress in other health sectors – e.g. to measure rates of improvement in the maternal mortality ratio. Use of such quantitative measures of progress allow policy-makers to make evidence-based decisions and provide the human rights community and others with an objective method for country comparison. Our results indicate that, in many countries, the progress being made towards universal access to improved drinking water and sanitation is far from the maximum achievable. The lack of relationship between the normalized rates of change and the nine national indicators that we investigated is important – particularly with respect to the economic variables. The finding that official development assistance is not correlated to our indicator of progress suggests that investment alone is not sufficient to ensure progress. In future studies, the effect on progress of additional variables that assess the enabling environment and governance should be investigated.

## References

[1] Resolution A/RES 64/292. The human right to water and sanitation. In: Sixty-fourth session, United Nations General Assembly, New York, 28 July 2010. Resolutions. New York: United Nations; 2010.

[2] Resolution A/HRC/RES 15/9. Human rights and access to safe drinking water and sanitation. In: 15th session of the Human Rights Council, United Nations General Assembly, New York, 30 September 2010. New York: United Nations; 2010.

[3] Draft outcome document of the United Nations summit for the adoption of the post-2015 development agenda. A/69/l.85. United Nations General Assembly. New York: United Nations; 2015.

[4] Indicators for monitoring the Millennium Development Goals. New York: United Nations Development Group; 2003.

[5] Progress on drinking water and sanitation: 2015 update. Geneva: World Health Organization; 2014. Available from: http://www.who.int/water_sanitation_health/monitoring/jmp-2015-update/en/ [cited 2015 Nov 25].

[6] Botting MJ, Porbeni EO, Joffres MR, Johnston BC, Black RE, Mills EJ. Water and sanitation infrastructure for health: The impact of foreign aid. Global Health. 2010;6(1):12. 10.1186/1744-8603-6-12 20670447 PMC2921361

[7] Wayland J. A drop in the bucket? The effectiveness of foreign aid in the water, sanitation, and hygiene (WASH) sector [Master of Arts thesis]. Washington: American University; 2014.

[8] Bain R, Luyendijk R, Bartram J. WIDER Working Paper No. 2013/88, Universal access to drinking water: the role of aid. Helsinki: UNU-Wider; 2013. Available from: http://www.wider.unu.edu/publication/universal-access-drinking-water [cited 2014 Aug 25].

[9] Wolf S. Does aid improve public service delivery? Rev World Econ. 2007;143(4):650–72. 10.1007/s10290-007-0126-8

[10] Wolf S. Water and sanitation for all? Rural versus urban provision. Int J Serv Econ Manag. 2009;1(4):358–70.

[11] Hopewell MR, Graham JP. Trends in access to water supply and sanitation in 31 major sub-Saharan African cities: an analysis of DHS data from 2000 to 2012. BMC Public Health. 2014;14(1):208. 10.1186/1471-2458-14-208 24576260 PMC3942065

[12] Country files [Internet]. Geneva: World Health Organization; 2014. Available from: http://www.wssinfo.org/documents/?tx_displaycontroller[type]=country_files [cited 2014 Feb 10].

[13] JMP’s method - deriving progress estimates [Internet]. Geneva: World Health Organization; 2014. Available from: http://www.wssinfo.org/definitions-methods/method/ [cited 2014 Mar 14].

[14] Fukuda-Parr S, Lawson-Remer T, Randolph S. An index of economic and social rights fulfillment: concept and methodology. J Hum Rights. 2009;8(3):195–221. 10.1080/14754830903110194

[15] Luh J, Baum R, Bartram J. Equity in water and sanitation: developing an index to measure progressive realization of the human right. Int J Hyg Environ Health. 2013 11;216(6):662–71. 10.1016/j.ijheh.2012.12.007 23333082

[16] Wilson PW. FEAR: frontier efficiency analysis with R [R package version 2.0]. Clemson: Clemson University; 2013.

[17] R: a language and environment for statistical computing. Vienna: R Development Core Team; 2011. Available from http://www.r-project.org/ [cited 2015 Jun 19].

[18] World development indicators [Internet]. Washington: World Bank; 2014. Available from: http://data.worldbank.org/data-catalog/world-development-indicators [cited 2014 Mar 14].

[19] Worldwide governance indicators [Internet]. Washington: World Bank; 2014. Available from: http://info.worldbank.org/governance/wgi/index.aspx [cited 2014 Mar 14].

[20] Query wizard for international development statistics [Internet]. Paris: Organisation for Economic Co-operation and Development; 2014. Available from: http://stats.oecd.org/qwids/ [accessed 14 Mar 2014].

[21] World population prospects: the 2012 revision [Internet]. New York: United Nations; 2014. Available from: http://esa.un.org/unpd/wpp/*DVD* [cited 2014 Mar 14].

[22] Barro-Lee educational attainment dataset [Internet]. Seoul: Robert J Barro and Jong-Wha Lee; 2013. Available from: http://www.barrolee.com/ [cited 2014 Mar 14].

[23] Human Development Index [Internet]. New York: United Nations Development Programme; 2014. Available from: http://hdr.undp.org/en/data [cited 2014 Mar 14].

[24] UN-water global annual assessment of sanitation and drinking water 2010 report. Geneva: World Health Organization; 2010.

[25] UN-water global analysis and assessment of sanitation and drinking-water 2014 report. Geneva: World Health Organization; 2014.

[26] UCDP/PRIO armed conflict dataset [Internet]. Uppsala: Uppsala Universitet; 2014. Available from: http://www.pcr.uu.se/research/ucdp/datasets/ucdp_prio_armed_conflict_dataset/ [cited 2015 Feb 11].

[27] Onda K, Crocker J, Kayser GL, Bartram J. Country clustering applied to the water and sanitation sector: a new tool with potential applications in research and policy. Int J Hyg Environ Health. 2014 3;217(2-3):379–85. 10.1016/j.ijheh.2013.07.017 24054545 PMC3946906

[28] Wilson PW. Detecting outliers in deterministic nonparametric frontier models with multiple outputs. J Bus Econ Stat. 1993;11:319–23.

[29] Andrews DF, Pregibon D. Finding the outliers that matter. J R Stat Soc, B. 1978;40:85–93.

